# Microfluidic Reactors for Carbon Fixation under Ambient-Pressure Alkaline-Hydrothermal-Vent Conditions

**DOI:** 10.3390/life9010016

**Published:** 2019-02-01

**Authors:** Victor Sojo, Aya Ohno, Shawn E. McGlynn, Yoichi M.A. Yamada, Ryuhei Nakamura

**Affiliations:** 1 RIKEN Center for Sustainable Resource Science, 2-1 Hirosawa, Wako, Saitama 351-0198, Japan; aohno@riken.jp (A.O.); mcglynn@elsi.jp (S.E.M.); ymayamada@riken.jp (Y.M.A.Y.); ryuhei.nakamura@riken.jp (R.N.); 2 Systems Biophysics, Ludwig-Maximilian University of Munich, Munich 80799, Germany; 3 Institute for Advanced Study, Berlin. Wallotstr. 19, Berlin 14193, Germany; 4 Earth-Life Science Institute, Tokyo Institute of Technology, 2-12-1 Ookayama, Meguro-ku, Tokyo 152-8550, Japan; 5 Blue Marble Space Institute of Science, Seattle, WA 98154, USA

**Keywords:** origin of life, abiogenesis, carbon fixation, hydrothermal vents, electrochemistry, reduction

## Abstract

The alkaline-hydrothermal-vent theory for the origin of life predicts the spontaneous reduction of CO_2_, dissolved in acidic ocean waters, with H_2_ from the alkaline vent effluent. This reaction would be catalyzed by Fe(Ni)S clusters precipitated at the interface, which effectively separate the two fluids into an electrochemical cell. Using microfluidic reactors, we set out to test this concept. We produced thin, long Fe(Ni)S precipitates of less than 10 µm thickness. Mixing simplified analogs of the acidic-ocean and alkaline-vent fluids, we then tested for the reduction of CO_2_. We were unable to detect reduced carbon products under a number of conditions. As all of our reactions were performed at atmospheric pressure, the lack of reduced carbon products may simply be attributable to the low concentration of hydrogen in our system, suggesting that high-pressure reactors may be a necessity.

## 1. Introduction

From its very start, life required reduced organic molecules. In a minimalistic scenario for abiogenesis (i.e. the emergence of life), one source of such molecules was the reduction of CO_2_, in a process overall similar (and potentially homologous) to the modern enzyme-facilitated pathways of extant autotrophic cells [1,2,3,4,5,6,7,8]. A number of reducing agents (i.e. sources of electrons) for reducing CO_2_ were possible on the early Earth, but multiple reasons make hydrogen (H_2_) a good candidate. This is discussed at length elsewhere (see [5,8,9,10] and references therein), but two points are worth mentioning here, namely: (1) hydrogen is formed spontaneously in the Earth’s crust via the “serpentinization” of ultramafic-rock minerals such as olivine [9,11,12,13]; and (2) it is used to reduce CO_2_ by members of both archaea and bacteria, in their respective versions of the Wood–Ljungdahl (WL) or acetyl Co-A pathway [5,14,15].

This process results in CO_2_ fixation and the production of ATP within a single, linear, metabolic pathway [5,16,17], so it has been suggested as a potential candidate for the metabolism of the last universal common ancestor (LUCA). However, one problem with extrapolating this scenario towards the origin of life is that, while the overall pathway of carbon fixation via the WL pathway is exergonic, the initial reaction between H_2_ and CO_2_ is not spontaneous under standard abiotic conditions [18]. Confirming these thermodynamic predictions, numerous experimental electrochemical results show that CO_2_ reduction is indeed disfavored under most observational conditions, requiring overpotentials of at least 180 mV in order to overcome the initial endergonic steps [19].

However, under putative ancient alkaline-vent conditions, CO_2_ would have been dissolved in slightly acidic ocean waters (pH 5~7), whereas H_2_ would have been a product of serpentinization, emanating as part of the efflux of the alkaline vent, itself rich in OH^—^ (pH 9~12). 

**Figure 1:**
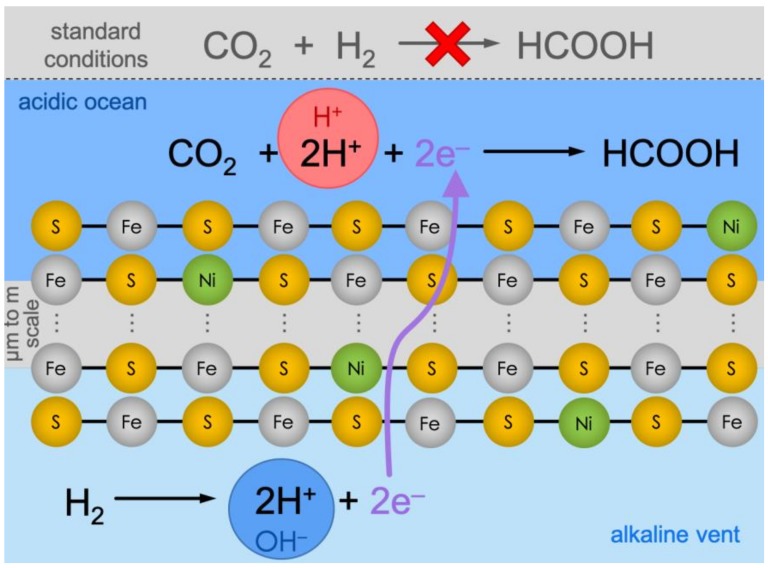
Under standard conditions (top, grey background), CO_2_ reduction with H_2_ is not viable thermodynamically. Conversely, in alkaline vents, the reaction is hypothetically split into two halves, effectively producing an electrochemical cell. On the alkaline-vent side (bottom, light-blue background), the oxidation of H_2_ to H^+^ is favored because of the alkaline pH in the vent fluid (symbolized by the blue circle with “OH^—^”). The electrons would travel through the micrometer-to-meter-scale catalytic Fe(Ni)S precipitate network and meet CO_2_ at the ocean side (dark-blue background), where the relatively acidic pH (red circle with “H^+^”) would favor the reduction and protonation towards formic acid (HCOOH).

The geologically sustained pH difference across the vent minerals provided an additional electrochemical driving force, potentially circumventing the lack of reducing power of H_2_ for CO_2_ reduction [19]. The reduction of CO_2_ to formic acid (HCOOH, its first 2-electron reduction product) involves protonation, so it would have been favored in acidic ocean waters. In turn, the oxidation of H_2_ releases protons (H^+^), which would have been favored in the alkaline waters that contained the dissolved H_2_ [20]. The two fluids would have been separated by the mineral precipitates of the vent, which included iron (and nickel) sulfides (Fe(Ni)S) as well as silicates (as reviewed in [21]). Reduced on the inside and oxidized on the outside, a situation analogous to an electrochemical cell would have existed between the two sides. The electrons released from H_2_ would then hypothetically travel through the electrically conductive Fe(Ni)S network [19], and drive the reduction of CO_2_ on the other side (Figure 1). This contrast between the pH of the two solutions matches the polarity of modern cells, and it has been suggested as a potential driver of the origins of both membrane bioenergetics and carbon fixation [6,7,8,20,22,23,24].

The pH gradients have also recently been shown to hold in the microscale, at up to six pH‑unit differences [25], suggesting the potential of microfluidic devices to study the reduction of CO_2_ with H_2_ under these conditions.

The immobilization of a catalytic boundary by the meeting of two fluids has been demonstrated using a microfluidic reactor [26], so we envisioned that this methodology could be applied to the formation of catalytic Fe(Ni)S clusters at the interface, elaborating on previous results [25]. This effectively mimics the ancient alkaline-vent conditions by the in-situ creation of an electrochemical cell between the oxidized CO_2_ in the acidic-ocean side and the reduced H_2_ in the alkaline-vent side (Figure 1).

Here, we present preliminary results in our study of the potential reduction of CO_2_ with H_2_. We used microfluidics to simulate the mixing of oceanic and serpentinizing fluids under the putative conditions of ancient alkaline hydrothermal vents—although notably at atmospheric pressure.

**Figure 2:**
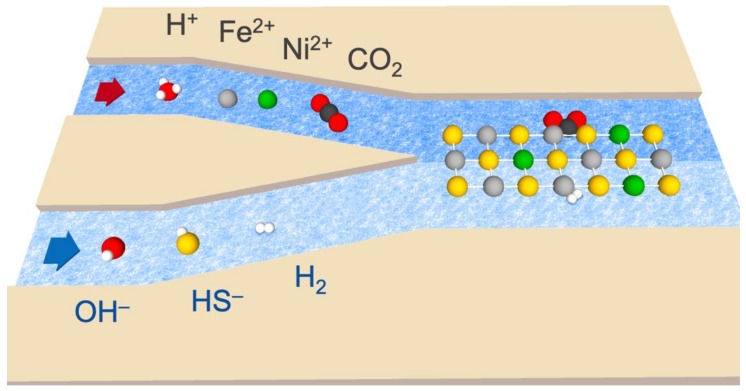
Diagram of the reaction system, depicting the input of acidic fluid (top, left half) containing H^+^, Fe^2+^, Ni^2+^, and dissolved CO_2_. The alkaline fluid (bottom, left half) contained OH^—^, HS^—^, and dissolved H_2_. Upon meeting, the fluids form Fe(Ni)S precipitates (represented by the reticulation in the right half), which may serve as catalysts for the indirect redox reaction between H_2_ and CO_2_.

We simulated the two sides of the vent system by mixing fluids containing combinations of Fe^2+^/Ni^2+^ and CO_2_ for the acidic-ocean side; whereas the alkaline-vent simulant contained HS^—^ and bubbled H_2_ (Figure 2).

## 2. Methods

The microfluidic reactor systems were assembled using custom chips with a Y-shape design, etched from glass by the Institute of Microchemical Technology Co., Ltd. The channels in the system were half-pipes with a width of 100 ± 2.5 µm and a maximum depth of 40 ± 1 µm.

At one tip of the “Y”, we input the analog of the acidic-ocean fluid, with the alkaline-vent analog being input at the other tip (as summarized in the diagram of Figure 2). The compositions of the fluids, presented in Table 1 and detailed in the Results, are similar to those reported elsewhere [10].

Because of the sensitivity of Fe^2+^ to oxygen in air, and in order to mimic the anoxic Hadean conditions at the origin of life more closely, the water in all of the experiments was de-gassed by boiling for 5 min and then cooling under constant argon bubbling for 30 min. The salts were weighed and then kept as solids under positive pressure of argon. The necessary amounts of de-aerated water were added, and the solutions bubbled with argon for another 15 minutes, with the exception of the H_2_-containing solutions, which were bubbled with H_2_. The final pH of the H_2_-containing solution was ~11, whereas that of the carbonic solution was ~6. Gas-tight syringes were then filled (all with a maximum volume of 1 mL, from Hamilton USA).

**Figure 3:**
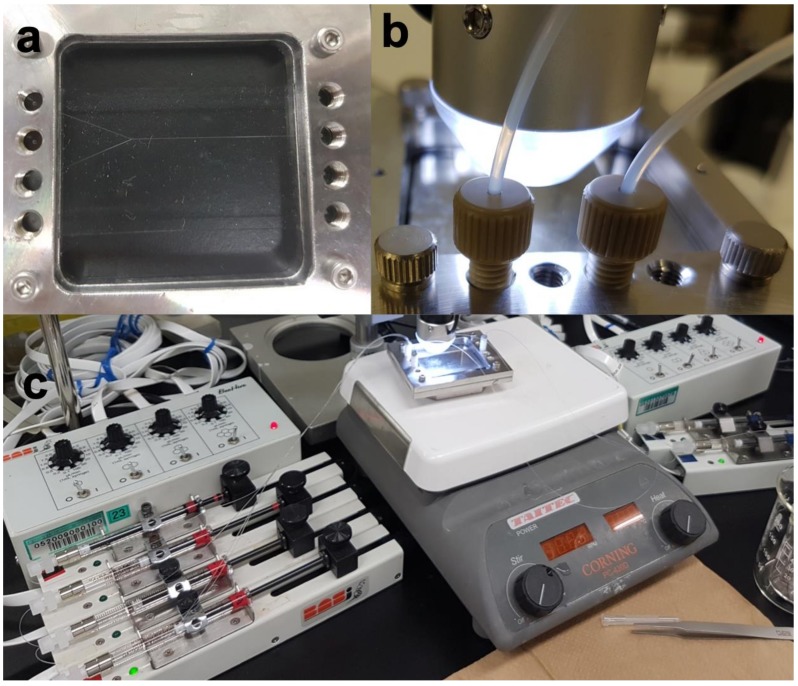
Reactor setup. (**a**) Microfluidic Y-shaped reactor chip with ~100 µm‑width channels, fitted into a stainless-steal holder with screw-in inlets. (**b**) The two inlets adjusted into position (bottom). A microscope (middle and top) was used to follow the precipitation reaction. (**c**) Left and far right: BASi drive controller and syringe pumps with Hamilton syringes. Center: reaction chip on the heating plate, with a USB microscope adjusted on top to follow the precipitation.

The full system setup is presented in Figure 3. The simulants of the acidic-ocean and alkaline-vent fluids were driven into the system using syringe pumps at adjustable flow rates, generally between 0.2 and 20 µL/min. The pumps were modular BabyBee Syringe Drive units from Bioanalytical Systems Inc. (BASi, West Lafayette, IN, USA), regulated by a BeeHive Syringe Drive Controller, also from BASi. The temperature was measured using an infrared thermometer, and regulated using a standard heating plate. A stainless-steel block was laid directly onto the heating plate, with the glass reactor laid on top of the block (Figure 3c, middle). The reactor chip was held in a custom-made stainless-steel casing (Figure 3a–c). The formation of the precipitates was followed using an inline USB microscope (Figure 3b, middle) connected to a standard laptop computer.

Reaction blanks were taken by running water through the chip on both inlets, after the precipitation reaction had taken place, but before adding any CO_2_/NaHCO_3_.

NMR spectra (^1^H and ^13^C) were determined using a JEOL spectrometer with a 600 MHz magnet.

## 3. Results

To facilitate microfluidic mixing and simulate the mixing of alkaline-vent and oceanic fluids, we replicated previous concentrations [10] and separated each of the fluids into independently controlled gas-tight syringes. For the acidic side, three inflows were used (see Table 1 for further details), as follows:

- De-aerated water (to achieve parallel flow and take sample blanks).
- FeCl_2_ (50 mM) and NiCl_2_ (5 mM).
- De-aerated water bubbled with CO_2_ (at atmospheric pressure), or alternatively dissolved NaHCO_3_ (100 mM), acidified with HCl (1 M) to pH 6.

Conversely, the two alkaline-side syringes contained the following fluids:

- De-aerated water.
- De-aerated water with Na_2_S (10 mM), K_2_HPO_4_ (10 mM), and Na_2_Si_3_O_7_ (10 mM), bubbled with H_2_ (at atmospheric pressure), and at a final pH of ~11.

**Figure 4:**
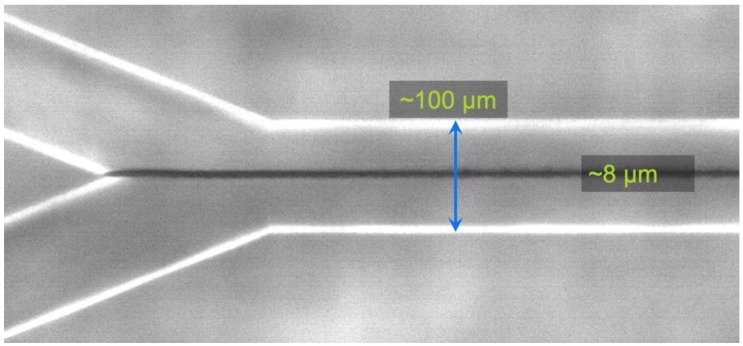
Precipitation of Fe(Ni)S at the interface between the acidic and alkaline fluids. By replacing the flow of either (or both) the sulfide or the metals with water, the precipitate could be kept arbitrarily thin.

After attaining parallel flow by letting water run from both inlets for 20 min, the mixing of the metal and sulfide fluids produced a thin dark precipitate at the interface. By changing either or both of the two fluids back to the water syringes, the thickness of the precipitate could be closely controlled (Figure 4).

Once the precipitates were formed at the interface, the inflows were swapped to the respective syringes containing CO_2_ and H_2_ (which, in the latter case, was the same as that for the sulfide in most of our reactions). The reaction conditions were controlled to last between 30 min and 24 h, and temperatures between laboratory conditions (~25 °C) and 70 °C (Table 1 and Methods).

**Figure A1:**
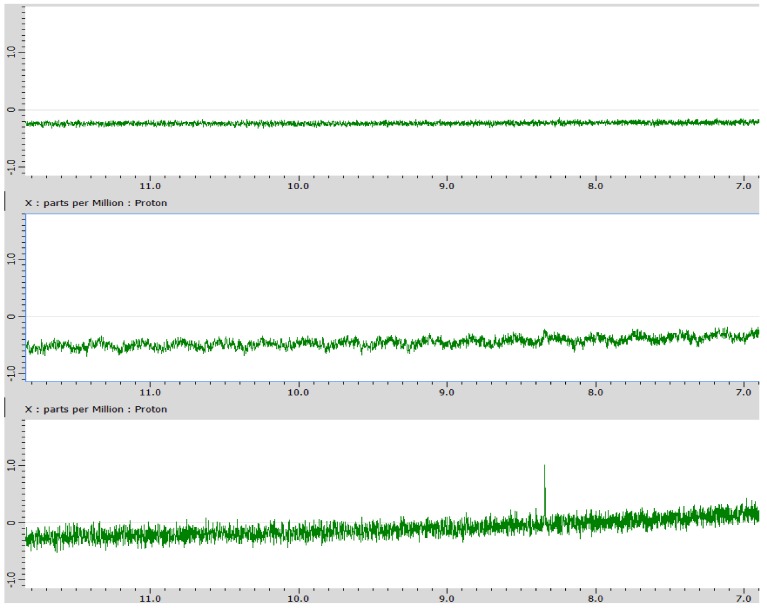
^1^H spectrum of dissolved NaHCO_3_ (top), reaction blank (middle), and reaction efflux (bottom). The reaction efflux shows a peak in the formic acid region, slightly over 8.3 ppm.

**Figure A2:**
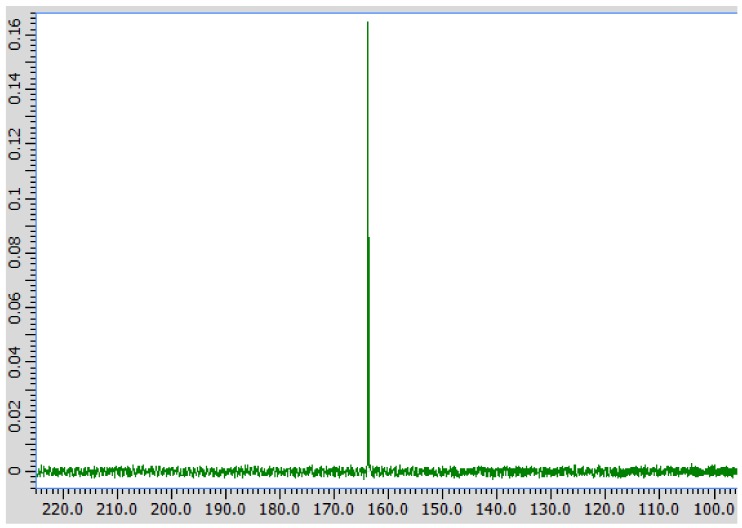
^13^C spectrum of reaction efflux, showing a peak in the formic acid region (~164 ppm).

**Figure A3:**
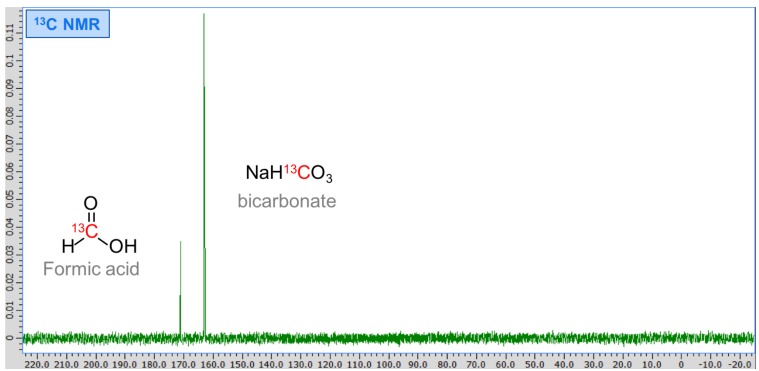
^13^C spectrum of reaction efflux with added ^13^C-labelled formic acid. Instead of a single increased peak, two peaks are visible, thus revealing that formic acid was not produced and the peak to the right corresponds to unreacted bicarbonate in the efflux.

**Figure A4:**
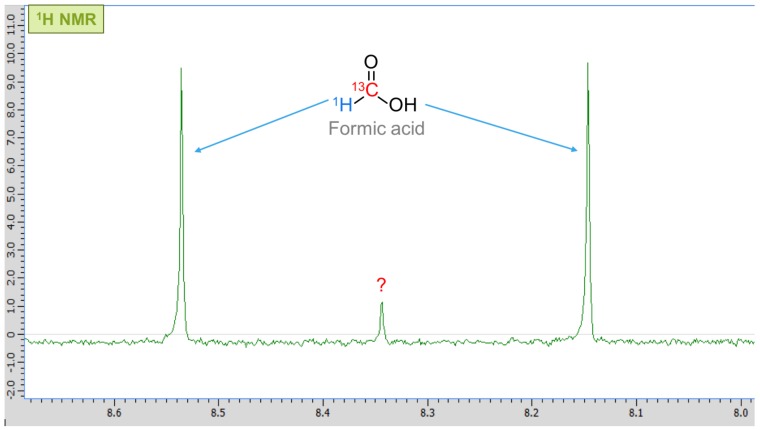
Adding ^13^C-labelled formic acid gives a doublet in the ^1^H spectrum, indicated by the arrows. The peak in the middle (~8.34 ppm) remains unidentified.

Analysis using ^1^H-NMR showed a peak in the formic acid region (~8.3 ppm, Figure A1). To assess whether the peak in this result was indeed due to formic acid, ^13^C‑labelled sodium bicarbonate (NaH^13^CO_3_), acidified to pH 6, was used. A peak in the formic-acid region (~164 ppm) of the ^13^C-NMR spectrum (Figure A2) was however shown to correspond to unreacted NaH^13^CO_3_ itself. This was confirmed by spiking with ^13^C‑labelled formic acid (H^13^COOH, Figure A3). The ^1^H spectrum for ^13^C‑labelled formic acid should show a splitting of the original singlet into a doublet. However, the doublet is not observed in the reaction efflux with NaH^13^CO_3_, appearing only when ^13^C‑labelled formic acid was added externally (Figure A4). The original peak at ~8.3 ppm in the ^1^H spectrum remains unidentified.

Overall, we did not detect reduced carbon products under the conditions that we tested. Varying conditions (Table 1), including concentrations, thickness of the precipitates, reaction temperature, reaction times, or doping the Fe(Ni)S precipitates with heteroatoms such as Mo(VI), produced no detectable difference.

## 4. Discussion

We aimed to probe the reduction of CO_2_ with H_2_ under putative ancient alkaline-hydrothermal-vent conditions.

In contrast to previous work [10], in which formate and formaldehyde were reported, we do not detect any soluble reduced carbon products under our experimental conditions. A separate set of experiments conducted with a larger-scale system modeled after previous work [27] also failed to yield detectable CO_2_ reduction (Chang and McGlynn, unpublished).

In view of this, it is important to stress that our experimental conditions were not exhaustive, and they failed to replicate alkaline vents in at least one crucial aspect—pressure.

The solubility of hydrogen is extremely low at ambient pressures, and decreases sharply as temperature rises towards the boiling point of water [28]. We bubbled H_2_ at atmospheric pressure, prior to the reaction, and did not continue bubbling during the reaction once the desired volume was stored in a gas-tight syringe. Since the H_2_-containing fluid in our experiments is at pH 11 and the CO_2_-containing fluid at pH 6, 1 bar of H_2_ at room temperature is predicted to be sufficient to reduce CO_2_, a reaction whose favorability would be enhanced with greater pressure as a result of the gain in concentrations [29,30]. The magnitude of the overpotential needed to overcome any kinetic barriers however remains unknown, and it is possible that high-pressure reactors are a necessity to overcome these barriers and evaluate the possibility of H_2_-powered reduction of CO_2_ under alkaline-vent conditions. Similarly, continuous bubbling of H_2_ (as in previous work [10]) may be necessary, given the high volatility of H_2_ gas.

Notably, recent results show that reduced carbon products are undetectable under similar reaction conditions using pure metals as catalysts [31,32], instead staying bound to the catalysts until concentrated KOH is used to remove them. It is therefore plausible (although it would need to be shown) that any reduced products that we may have formed remained bound to the Fe(Ni)S precipitates. We also note that we did not investigate potential gas-phase products in our study, leaving these as possibilities to be explored.

The electrical potential from the pH gradient under alkaline-vent conditions has been measured at the microscale [25], but it remains to be shown that it can indeed drive otherwise unfavorable redox reactions. Thus, to test the validity of the electrochemical-cell concept—irrespective of its relevance to the origin of life—further (potentially less geologically relevant) experiments could include using stronger reducing and oxidizing agents, at varying concentrations. The utilization of high-pressure reactors to achieve higher concentrations of dissolved gas may be especially important to test for H_2_-driven reductions.

Broadly, our scheme for overcoming the exergonic steps of carbon reduction relies on the separation of solutions at different conditions coupled to the ability to transfer electrons, which is not unique to pH gradients at alkaline vents—thermal gradients and reducing agents other than H_2_ are also possible drivers of reduction [29,30].

The alkaline-hydrothermal-vent theory has come under criticism in recent years [33,34,35]. These issues have been addressed elsewhere [36], but it is important to note here that we do not see our results as either disproving the alkaline-vent theory, or providing support for alternative theories for the origin of life. Most simply, they suggest that the catalysts that appropriately lower the kinetic barriers have not been implemented as of yet, or that more realistic conditions—crucially higher pressures, particularly for H_2_—need to be evaluated to more closely in order ascertain the viability of the hypotheses tested here. These are lines of enquiry that we are pursuing, as are other researchers in the field.

## Figures and Tables

**Table 1:** Conditions tested. The concentrations of NaHCO_3_ were inevitably lower than shown after acidification to pH 6 with 1 M HCl. In alternative experiments we used CO_2_ bubbled at atmospheric pressure instead of NaHCO_3_.

ACIDIC-SIDE CONCENTRATIONS		ALKALINE-SIDE CONCENTRATIONS	
**[FeCl_2_]**	50 mM	[Na_2_S]	10 and 100 mM
**[NiCl_2_]**	0 and 10 mM	[K_2_HPO_4_]	10 mM
**[NaHCO_3_]**	10, 50, and 100 mM (acidified to pH ~6)	[Na_2_Si_3_O_7_]	0 and 10 mM
		[Na_2_MoO_4_]	0 and 1 mM
**CO_2_**	Bubbled at atmospheric pressure (final pH ~6)	[H_2_]	Bubbled at atmospheric pressure (final pH ~11)
**OTHER CONDITIONS**			
**Reaction durations**	½, 1, 2, 5, 12, and 24 h	Temperature	~25 (room), 40, 50, 60, and 70 °C
